# Identifying novel phenotypes of elevated left ventricular end diastolic pressure using hierarchical clustering of features derived from electromechanical waveform data

**DOI:** 10.3389/fcvm.2022.980625

**Published:** 2022-09-23

**Authors:** Timothy Burton, Shyam Ramchandani, Sanjeev P. Bhavnani, Rola Khedraki, Travis J. Cohoon, Thomas D. Stuckey, John A. Steuter, Frederick J. Meine, Brett A. Bennett, William S. Carroll, Emmanuel Lange, Farhad Fathieh, Ali Khosousi, Mark Rabbat, William E. Sanders

**Affiliations:** ^1^CorVista Health (Analytics For Life Inc., d.b.a CorVista Health) Toronto, Toronto, ON, Canada; ^2^Scripps Clinic Division of Cardiology, San Diego, CA, United States; ^3^Cone Health Heart and Vascular Center, Greensboro, NC, United States; ^4^Bryan Heart, Lincoln, NE, United States; ^5^Novant Health New Hanover Regional Medical Center, Wilmington, NC, United States; ^6^Jackson Heart Clinic, Jackson, MS, United States; ^7^Cardiology Associates of North Mississippi, Tupelo, MS, United States; ^8^Division of Cardiology, Loyola University Medical Center, Maywood, IL, United States; ^9^CorVista Health, Inc., Washington, DC, United States; ^10^University of North Carolina at Chapel Hill, Chapel Hill, NC, United States

**Keywords:** machine learning, risk stratification, left ventricular filling pressures, artificial intelligence, digital health

## Abstract

**Introduction:**

Elevated left ventricular end diastolic pressure (LVEDP) is a consequence of compromised left ventricular compliance and an important measure of myocardial dysfunction. An algorithm was developed to predict elevated LVEDP utilizing electro-mechanical (EM) waveform features. We examined the hierarchical clustering of selected features developed from these EM waveforms in order to identify important patient subgroups and assess their possible prognostic significance.

**Materials and methods:**

Patients presenting with cardiovascular symptoms (*N* = 396) underwent EM data collection and direct LVEDP measurement by left heart catheterization. LVEDP was classified as non-elevated ( ≤ 12 mmHg) or elevated (≥25 mmHg). The 30 most contributive features to the algorithm output were extracted from EM data and input to an unsupervised hierarchical clustering algorithm. The resultant dendrogram was divided into five clusters, and patient metadata overlaid.

**Results:**

The cluster with highest LVEDP (cluster 1) was most dissimilar from the lowest LVEDP cluster (cluster 5) in both clustering and with respect to clinical characteristics. In contrast to the cluster demonstrating the highest percentage of elevated LVEDP patients, the lowest was predominantly non-elevated LVEDP, younger, lower BMI, and males with a higher rate of significant coronary artery disease (CAD). The next adjacent cluster (cluster 2) to that of the highest LVEDP (cluster 1) had the second lowest LVEDP of all clusters. Cluster 2 differed from Cluster 1 primarily based on features extracted from the electrical data, and those that quantified predictability and variability of the signal. There was a low predictability and high variability in the highest LVEDP cluster 1, and the opposite in adjacent cluster 2.

**Conclusion:**

This analysis identified subgroups of patients with varying degrees of LVEDP elevation based on waveform features. An approach to stratify movement between clusters and possible progression of myocardial dysfunction may include changes in features that differentiate clusters; specifically, reductions in electrical signal predictability and increases in variability. Identification of phenotypes of myocardial dysfunction evidenced by elevated LVEDP and knowledge of factors promoting transition to clusters with higher levels of left ventricular filling pressures could permit early risk stratification and improve patient selection for novel therapeutic interventions.

## Introduction

An elevated left ventricular end diastolic pressure (LVEDP), indicative of increased left-sided filling pressures of the heart, represents a critical and sensitive measurement used to aid in the identification of impending as well as decompensated heart failure (HF) (1–3). Cardiac performance, with regard to ventricular contractility, is one critical determinant of elevated LVEDP; however, ejection fraction (EF) alone rarely elucidates the accurate clinical status of a heart failure patient (4). Patients with diminished ventricular function are the cohort most frequently encountered with elevated LVEDP, but an increased filling pressure may be the manifestation of multiple myocardial disease states including cardiomyopathies of ischemic, constrictive, restrictive, or valvular origin (5). Thus, higher filling pressures typically precede clinical deterioration in heart failure with reduced ejection fraction (HFrEF) as well as heart failure with preserved ejection fractions (HFpEF) (6). An elevated LVEDP does not indicate a specific diagnosis but provides important information that serves as a guide regarding the need for further evaluations or testing and affords data necessary for the development of an appropriate patient care plan. In addition, elevated LVEDP is predictive of morbidity and mortality (7–11).

Awareness of possible impending or new onset heart failure (HF) as indicated by an elevation in LVEDP may aid in diagnosis and risk stratification of the patient and help inform optimal clinical care pathways. The available therapeutic modalities for HF have doubled in the last decade and new pharmacologic agents for the treatment of hypertrophic cardiomyopathy (HCM) and infiltrative diseases, such as amyloid, conditions associated with elevated LVEDP, are rapidly emerging (12, 13). However, there exists limited information regarding the risks of transition from early asymptomatic or mildly symptomatic stages (Stages A to B) to later overtly symptomatic stages (Stages C-D), thus complicating initiation of drug interventions at the times when such therapies might prove most beneficial (14).

Risk stratification in heart failure remains a significant challenge despite the development of multiple scoring models, many of which are designed to predict survival with a few attempting to predict future morbidity (15–19). These scoring models were primarily derived from highly selected cohorts of patients, with an established diagnosis of heart failure, recruited for randomized clinical trials occurring over the last several decades (20). Most have required input data that includes echocardiographic determination of EF, multiple blood analyses (recently biomarkers), and a significant number of clinical parameters (15–21). Predicting outcomes in those with known moderate to severe disease allows modification and testing of current therapies but provides little information that might permit interventions at early stages of heart failure to prevent progression. Machine learning affords the opportunity with a single rapid test to detected features of similarities between groups with early stage LVEDP elevation and those not yet manifesting hemodynamic changes (22). ML clustering techniques that identify such features facilitate the development and future validation of new risk models.

The clinical diagnosis of HF encompasses a broad and heterogeneous population of patients with complex pathophysiology, various etiologic mechanisms, and diverse genetic triggers. Unsupervised clustering analysis has permitted phenotyping in cardiomyopathies identifying subgroups which may have similar mechanisms and outcomes (23, 24). Clustering is a useful approach to detect patterns in variables that aid in decrypting the heterogeneity present in datasets (24, 25). To date, the evaluation of cardiomyopathies based on clustering has focused on patient with diagnosed HF and those recently or in the past hospitalized for the HF, clearly in later stages of the disease (23, 24). In this trial, we recruited a cohort with new onset symptoms and no previously known HF. The goal was to detect changes in the features that differentiate between clusters, which might indicate the risk of transition from one cluster to an adjacent cluster with a higher prevalence of elevated LVEDP. Identification of distinct subtypes of myocardial dysfunction evidenced by elevated LVEDP and knowledge of factors that promote transition to clusters with higher levels of left ventricular filling pressures could permit early risk stratification and improve patient selection for novel therapeutic interventions, thus facilitating precision medicine.

## Methods

### Features for clustering

**Figure 1:**
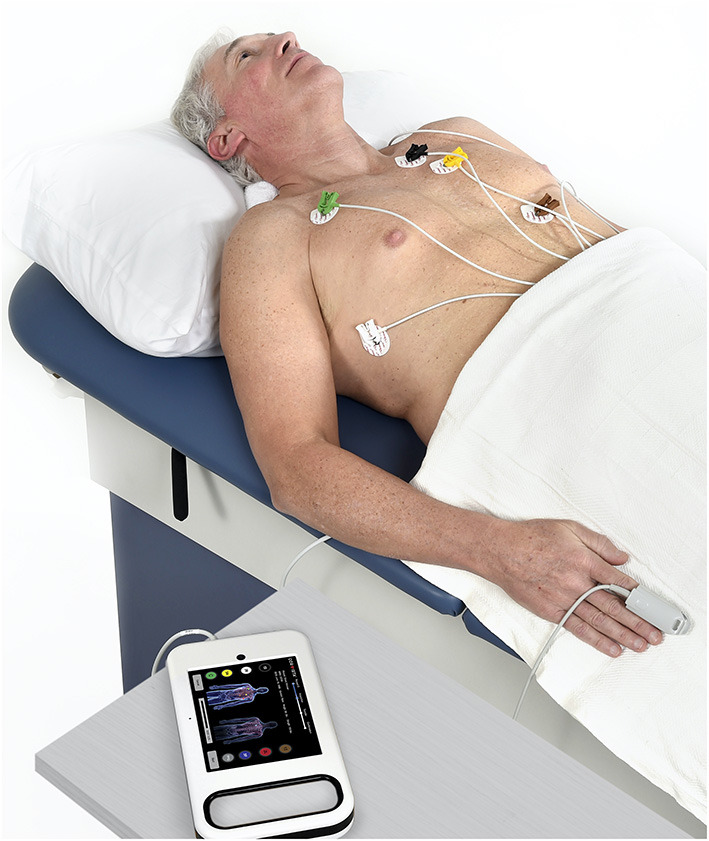
Signal acquisition using the CorVista Capture, with the electrodes placed on the torso (electrode on the back not visible), and the PPG clip placed on the finger.

**Figure 2:**
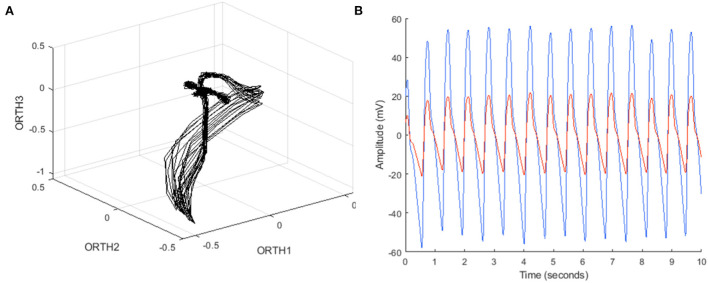
**(A)** Example OVG data in phase space, with coordinates from each bipolar channel (ORTH1, ORTH2, ORTH3) represented as a three-dimensional coordinate in that space, and **(B)** example PPG data in the time domain, containing both red and infrared time series.

An algorithm was previously developed to predict LVEDP elevation status based on manually engineered features calculated from CorVista Capture signals (22). The signal acquisition modality was previously described (26), but briefly, is the simultaneous acquisition of orthogonal voltage gradient (OVG) data *via* electrodes placed on the torso, and photoplethysmogram using transmission of red and infrared light *via* a clip placed on the finger. The acquisition configuration is shown in Figure 1. The OVG signal is related to the electrocardiogram (ECG) signal in that both measure the voltage changes within the myocardium that occur during the cardiac cycle, but OVG differs from ECG in its three-dimensional perspective of the heart, as well as its high frequency bandwidth (Figure 2).

The contribution of these features to the LVEDP algorithm were assessed using a permutation analysis, enabling the ranking of the features from most to least contributive. The permutation analysis was a generalization of the methodology first proposed by Breiman for use in Random Forests (27). The top 30 most contributive features were selected for use in this clustering exploration.

### Population

The clinical population analyzed in the present work were subjects with symptoms suggestive of cardiovascular disease who were referred to cardiac catheterization for the assessment of coronary artery disease using angiography. Specifically, the cohort was patients in whom the treating physician chose to measure the LVEDP during the catheterization. LVEDP measurement was performed using standard catheterization laboratory procedures. Subjects with mid-range LVEDPs were removed, preserving subjects with definitive LVEDP non-elevation of ≤ 12 mmHg and subjects with definitive LVEDP elevation of ≥25 mmHg. The subjects were enrolled within the CADLAD and IDENTIFY (Group 2) studies, the records for which are available on clinicaltrials.gov (NCT02784197 and NCT03864081, respectively). Both studies have identical inclusion and exclusion criteria and are multi-center in nature. CADLAD is closed, and IDENTIFY enrollment is ongoing. Finally, subjects without a signal passing a series of automated signal quality assessment tests (11%) were excluded. Signal quality assessment was previously described (26), but included quantification of powerline interference and excessive high-frequency content in the OVG signal, and the presence of jumps, dropouts, and railing in the PPG signal.

### Clustering

The features were pre-processed prior to clustering by applying the box-cox transformation to each feature individually, which is a monotonic power transform intended to approximate a normal distribution across the data (28). While deviations from normality occur frequently in realistic data, many statistical approaches rely on an assumption of normality, and therefore benefit from distribution transformations such as box-cox. For instance, nearest neighbor classification has been shown to improve considerably when the features are pre-processed using box-cox, as compared to classification using the raw feature values (29). While the intent of the present work is not nearest neighbor classification (or classification by any methodology), clustering similarly necessitates pairwise distance measurements across the dataset, and can therefore realize a similar benefit from box-cox. After box-cox transformation, the features were normalized using the z-score transformation to set the mean and standard deviation of each feature to zero and one, respectively.

Next, hierarchical clustering was applied to the processed data. While there exist a variety of clustering algorithms, hierarchical clustering was chosen to facilitate visual discovery of the appropriate numbers of clusters from the clustering result, rather than requiring pre-specification of the number of clusters, or quantitative evaluation of the optimal number of clusters. Hierarchical cluster is agglomerative, meaning that initially each subject is initially its own cluster, then clusters are merged in an agglomerative manner, until the end state is reached where all the subjects belong to the same cluster. Therefore, a cluster merger strategy, also known as linkage, must be selected. Possibilities include “single” (smallest pairwise distance between the subjects in one cluster and the subjects in another), “complete” (the largest pairwise difference between the subjects in one cluster and the subjects in another), and “average” (the average pairwise difference between the subjects in one cluster and the subjects in another). Note that the linkages are equivalent when considering clusters containing only one subject. Given that average considers all subjects in both clusters, it was chosen for the present work. However, as described for all linkage methods, average linkage requires the definition of a distance metric between subjects, which are represented herein by the values of 30 features. A distance metric increases in magnitude as subjects become more dissimilar, and must meet specific mathematical criteria to quality as a proper metric (30). Correlation distance (more precisely, 1-Pearson correlation) was shown to generally perform well across a variety of datasets and clustering algorithms, and so was chosen herein (31).

The output of hierarchical clustering is a dendrogram, which is a visual representation of subjects beginning in their own clusters, and iterative merging of clusters until all the subjects belong to a single cluster. The dendrogram was inspected, and an appropriate number of clusters chosen based on both the structure of the dendrogram and the appropriate amount of data in each cluster.

## Results

A total of 396 subjects were included in the clustering, composed of 92 (23%) subjects with LVEDP≥25 mmHg and 304 (77%) subjects with LVEDP ≤ 12 mmHg. Of the top 30 features from the LVEDP algorithm included in the clustering, 22 were derived from the OVG signal and had an average rank in the contribution of 17, and eight were derived from the PPG signal with an average rank of 10.

**Figure 3:**
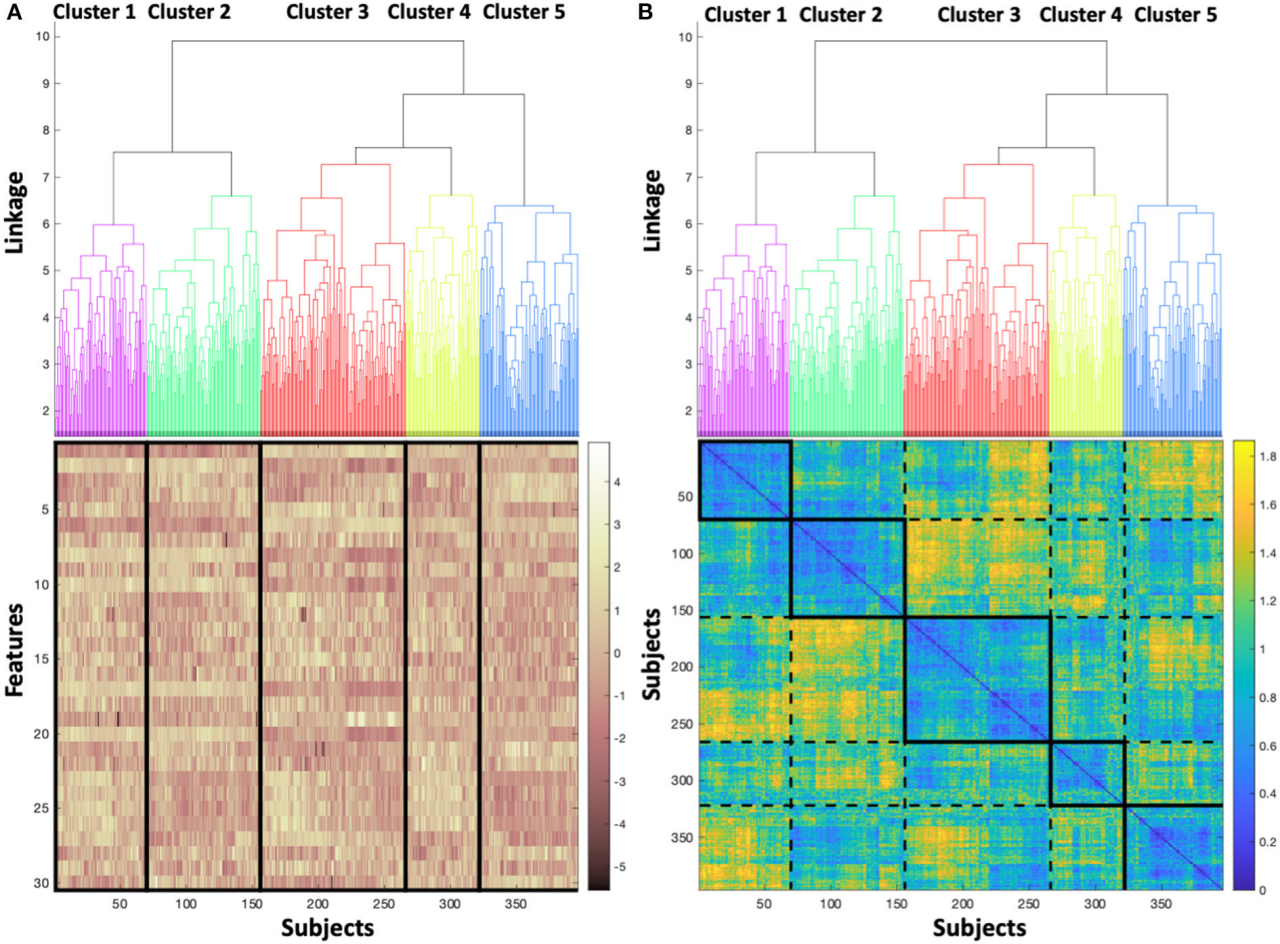
**(A)** Dendrogram colored to identify each cluster, associated to a heatmap visualizing the magnitude of the feature values for each subject, and **(B)** colored dendrogram associated with the pairwise distance matrix across the dataset, with bold boxes defining each cluster, and dotted lines delineating between adjacent clusters.

Figure 3 shows the dendrogram, with five clusters (labeled 1–5) chosen to segment the dataset, which for simplicity and recognition are colored (Purple=cluster 1, Green=cluster 2, Red=cluster 3, Yellow=cluster 4, Blue=cluster 5). In Figure 3A, the dendrogram is associated to a heatmap visualizing the magnitude of the feature values for each subject, with vertical lines delineating the boundary between adjacent clusters, and subjects aligning between the dendrogram and the heatmap. Banding in the heatmap that differs between clusters is a visual manifestation of the feature values differing across clusters. In Figure 3B, the dendrogram is associated to the pairwise distance matrix across the dataset, with the bold boxes along the diagonal defining each cluster, and the dotted lines delineating between adjacent clusters. Similar to Figure 3A, the subjects are aligned between the dendrogram and the distance matrix. As expected, the pairwise distances within clusters (i.e., within bolded boxes, occurring along the diagonal of the distance matrix) are low, showing that the distance between subjects within the same cluster is generally low. Therefore, subjects are cohesive within the clusters. The off-diagonal boxes defined by dashed lines in the distance matrix represent the distances between subjects not belonging to the same cluster, and as expected, exhibit larger distances on average than subjects belonging to the same cluster. Further, the distance between the cluster 1 (Purple) and cluster 2 (Green) is relatively low, as is supported by the dendrogram, which indicates that the next level of linkage would join these two clusters. Similarly, large distances in the heatmap are concentrated in the region comparing cluster 2 (Green) to cluster 3 (Red), which would not be joined together until the top linkage level, where all subjects are joined in a single cluster.

After the clustering was complete, clinical metadata was overlaid on the resultant clusters, as shown in Table 1. The overlaid clinical metadata can then be considered in conjunction with the observations from the feature clustering, as shown in Figure 3.

**Table 1:** Clusters demographics and measured parameters.

**Property**	**Purple**	**Green**	**Red**	**Yellow**	**Blue**
*N*	69	86	110	56	75
LVEDP>25	36.2%	22.1%	22.7%	26.8%	10.7%
LVEDP (mmHg)	17.8 ± 10.8	13.5 ± 8.5	13.9 ± 8.4	15.0 ± 9.9	14.2 ± 9.0
Age (years)	65.3 ± 9.6	62.9 ± 10.4	63.4 ± 9.2	63.7 ± 10.4	61.6 ± 11.6
BMI (kg/m^2^)	34.5 ± 7.6	31.0 ± 6.4	30.8 ± 7.2	32.0 ± 7.5	30.0 ± 6.0
Female	49.3%	40.7%	40.0%	42.9%	25.3%
Significant CAD*	40.6%	33.7%	38.9%	35.7%	44.0%
Diabetes	36.2%	32.6%	39.3%	34.9%	21.3%
Hypertension	78.3%	69.8%	76.8%	70.6%	68.0%
Hyperlipidemia	78.3%	75.6%	73.2%	72.5%	62.7%

^*^Significant CAD was defined as the presence of a >70% lesion or an FFR < 0.80, assessed during the same left heart catheterization procedure in which the LVEDP measurement was acquired.

Cluster 1, with the highest LVEDP of all clusters, is the most dissimilar from the cluster 5 in both the clustering and with respect to the clinical properties of the subjects in each cluster. In contrast to cluster 1, cluster 5 is predominantly non-elevated LVEDP, younger, lower BMI, male and has a higher rate of significant coronary artery disease. The most substantial feature differences between cluster 1 and cluster 5 are those calculated from the PPG signal. Given the gender and age differences between these clusters, a plausible explanation of the PPG differentiation may be the vasculature changes that occur in post-menopausal women.

Cluster 1 is closest in the clustering to cluster 2 (i.e., the next cluster that would be joined to cluster 1 in the dendrogram is cluster 2), with the second lowest LVEDP of all clusters. To explore the mechanism underlying the difference between these clusters, the feature values were normalized across the full dataset using z-score (i.e., mean set to zero, and standard deviation set to one), and the normalized values mapped on to the clusters. The feature values were averaged within cluster 1 and cluster 2, then the averages differenced across these two clusters. The largest differences between cluster 1 and cluster 2 were found in features extracted from the electrical data, and specifically those that quantify predictability and variability of the signal. There is low ability to predict the signal (occurring as high feature values) and high signal variability (occurring as high feature values) in the cluster 1, and the opposite in the cluster 2 (occurring with low feature values). The predictability functions by fitting a model to a portion of the OVG data and evaluating the performance of that model on the remaining data. Should the ability of the model to predict on the withheld data be poor, then the resultant error between the signal and the model will be high, resulting in a high feature value. The variability feature derives a representative cardiac cycle for the subject based on the acquired data and compares that representative cardiac cycle to each acquired cycle to calculate the disparity – should it be high, then the representative cycle cannot sufficiently capture the variability in the signal, which is then represented as a high feature value.

**Figure 4:**
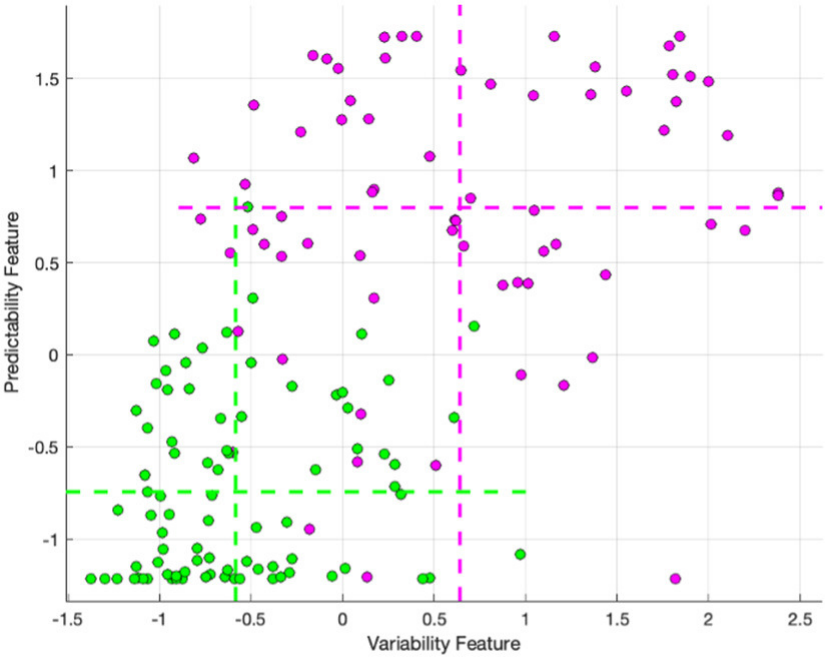
The variability and lack of predictability feature values for cluster 2 (Green) and cluster 1 (Purple), with the mean of each feature for each cluster marked with dashed lines.

The relationship between these features and clusters is shown in Figure 4, which visualizes the feature values for clusters 1 and 2, with the mean of each feature for each cluster marked with dashed lines. The mean predictability feature was −0.74 and 0.80, for cluster 2 and cluster 1, respectively. The mean variability feature was −0.58 and 0.64, for cluster 2 and cluster 1 respectively.

## Discussion

Machine-learned algorithms now permeate many facets of medical practice and the magnitude of these techniques' utility and their diverse applications are rapidly being adapted to challenges in cardiology (32, 33). Cluster analysis of datasets identifies patterns and trends by using the relationships between variables to uncover hidden structure (34). In the past, the heterogeneous nature of HF populations and the complexity of the pathophysiologic mechanisms operative in specific HF disease states have made identification of similar phenotypes difficult. The utilization of clustering analysis allows detection of small unique phenogroups by examining the similarities and differences among quantitative variables (34). This type of unsupervised ML has be applied to populations of HF including HFpEF (23, 35), HFrEF (36), and acute HF (37). These studies have elucidated phenotype clusters and demonstrated subsequent clinical outcomes of patients previously diagnosed with HF as well as those acutely admitted with that diagnosis. This approach is useful for risk stratification and prognostic prediction in later stages of the disease but does little for targeting early transition into pathophysiologic states at risk for progression to significant disease or to facilitate precision interventions at times when remodeling might be prevented.

In our trial, we identify novel phenotypes with varying prevalences of elevated LVEDP within a population presenting with new onset cardiovascular symptoms. Elevated LVEDP has served as a direct measurement of myocardial dysfunction as well as a marker indicative of pathophysiologic changes ultimately resulting in the remodeling observed in later stages of HF (38). The goal of this investigation was to analyze the phenotypes and determine if features within the clusters varied in a fashion that could be followed to predict transition to adjacent clusters with increased risk of LVEDP elevation.

This trial demonstrates five clusters resulting from the analysis of 396 subjects (Figure 3), composed of 92 (23%) subjects with LVEDP≥25 mmHg and 304 (77%) subjects with LVEDP ≤ 12 mmHg. The heatmap of the magnitude of feature values (Figure 3A) illustrates the similarity of cluster 1 and cluster 2 (purple and green) phenogroups with regard to each of the 30 features. The degree of similarity can be appreciated visually, with the density of yellow and brown being closest between cluster 1 and 2 (purple and green). When moving from cluster 1 (shown in column 1) to cluster 5 (column 5), the coloration representing feature values becomes progressively more discrepant to cluster 1.

Correspondingly, the pairwise distance matrix (Figure 3B) showing the distance between pairs of subjects yields a diagonal line that elucidates the basis behind the formation of each cluster. Each solid box on the diagonal line starting with cluster 1 (top left, column 1) and progressing down the diagonal to bottom right to arrive at cluster 5, indicates very similar distancing between points in each cluster (high intensity of blue coloring). The dotted rectangle boxes permit comparison between the clusters with reference to the distance between subjects. In addition, this provides confirmation of the appropriateness and accuracy of the resultant clusters.

Cluster 1 and the adjacent cluster 2 demonstrate the closest feature values, but interestingly, have the widest divergence of prevalence of elevated LVEDP. Their proximity suggests that modest changes in feature values could result in transition of a subject in cluster 2 to cluster 1, and consequently may result in increased risk of elevation in LVEDP.

Of the top 30 features from the LVEDP algorithm included in the clustering, 22 were derived from the OVG signal, and eight were derived from the PPG signal. We found that features quantifying predictability and variability exhibited the most substantial differences between the cluster with the highest rate of LVEDP elevation (cluster 1) and the adjacent cluster 2, which presented with the second lowest rate of LVEDP elevation across the clusters. This feature difference suggests that it may be possible to follow subjects initially belonging to the cluster 2 phenotype to determine whether the signal properties shift over time to increased variability and decreased predictability, indicating that they may be transitioning to the adjacent cluster 1, with the associated higher prevalence, and therefore risk of, LVEDP elevation. Validation of the described phenotypes can be achieved through clinical follow-up to detect outcomes. In addition, opportunities exist to further characterize the phenotypes by inclusion of supplementary clinical data, including biomarkers such as BNP.

## Conclusion

An approach to stratify the likelihood of movement between clusters and the possible progression of myocardial dysfunction for an individual patient could include changes in the features that differentiate these clusters; specifically, reductions in electrical signal predictability and increases in variability. Identification of distinct subtypes of myocardial dysfunction evidenced by elevated LVEDP and knowledge of factors that promote transition to clusters with higher levels of left ventricular filling pressures may permit early risk stratification and improve patient selection for novel therapeutic interventions thereby facilitating precision medicine.

## Data availability statement

The datasets presented in this article are not readily available because signals obtained by proprietary device, not yet FDA approved. Requests to access the datasets should be directed to wsanders@corvista.com.

## Ethics statement

The studies involving human participants were reviewed and approved by the Western Institutional Review Board, Inc. The patients provided their written informed consent to participate in this study. Written informed consent was obtained from the individual for the publication of any potentially identifiable images or data included in this article.

## Author contributions

Concept and design: SB, WS, TB, RK, and SR. Drafting of the manuscript: WS, TB, SB, MR, FF, AK, and EL. Statistical analysis: HG, EL, TB, AK, and FF. Literature search: WS, TB, FF, AK, SR, and MR. Obtained funding and final approval of the version to be published: WS. Administrative, technical, and material support: WS and TB. Supervision: WS. Acquisition, analysis, interpretation of data, and critical revision of the manuscript for important intellectual content: All authors. All authors contributed to the article and approved the submitted version.

## Funding

This study was supported by CorVista Health.

## Conflict of interest

CorVista Health funded the collection of subject data. Authors WS, HG, TB, FF, AK, EL, and SR are employees of CorVista Health. MR is a member of the Medical Advisory Board for CorVista Health. The remaining authors declare that the research was conducted in the absence of any commercial or financial relationships that could be construed as a potential conflict of interest.

## Publisher's note

All claims expressed in this article are solely those of the authors and do not necessarily represent those of their affiliated organizations, or those of the publisher, the editors and the reviewers. Any product that may be evaluated in this article, or claim that may be made by its manufacturer, is not guaranteed or endorsed by the publisher.
