# Supplementary material for: Identifying novel phenotypes of elevated left ventricular end diastolic pressure using hierarchical clustering of features derived from electromechanical waveform data
Source: Front Cardiovasc Med. 2022 Sep 23;9:980625. doi: 10.3389/fcvm.2022.980625 (PMC9539436; doi:10.3389/fcvm.2022.980625)
Supplement: Supplementary file 2 [file Table_1.DOCX]

| **Designation** | **Feature Domain** | **Description** |
| --- | --- | --- |
| A | Dynamics of the OVG and PPG signals | Features inspired by dynamical systems theory |
| B | Spectral properties of OVG and PPG signals | Features derived from power spectral and time-frequency analysis |
| C | Divergences of the OVG signal from subject-specific models | Features that quantify the divergence of the OVG signal from subject-specific models |
| D | Traditional time-domain features and variability of those features | Features that quantify traditional cardiac measurements, and their variability, in the OVG signal |
| E | Phase space | Features calculated using a phase space methodology (i.e., without time). |
| F | Photoplethysmographic time domain | Features that capture characteristics of the PPG in the time domain. |
